# Ga–Ni supported catalytically active liquid metal solutions (SCALMS) for selective ethylene oligomerization†

**DOI:** 10.1039/d1cy01146d

**Published:** 2021-11-15

**Authors:** Alexander Søgaard, Ana Luíza de Oliveira, Nicola Taccardi, Marco Haumann, Peter Wasserscheid

**Affiliations:** Friedrich-Alexander-Universität Erlangen-Nürnberg (FAU), Lehrstuhl für Chemische Reaktionstechnik (CRT) Egerlandstr. 3 91058 Erlangen Germany peter.wasserscheid@fau.de; Forschungszentrum Jülich GmbH, Helmholtz-Institute Erlangen-Nürnberg for Renewable Energy (IEK 11) Egerlandstr. 3 91058 Erlangen Germany

## Abstract

Non-precious metal supported catalytically active liquid metal solutions exhibit attractive performance in ethylene oligomerization. It is found for the Ga–Ni system on silica that the performance depends strongly on the applied Ga/Ni ratio. Ga-rich systems forming liquid alloys exhibit a far higher Ni-based catalytic activity than solid intermetallic compounds or Ni nanoparticles.

## Abstract 2

Non-precious metal supported catalytically active liquid metal solutions exhibit attractive performance in ethylene oligomerization.

## Graphical abstract

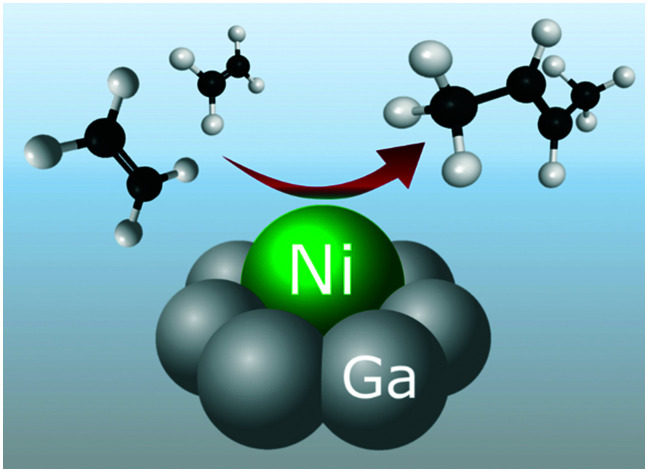

Supported catalytically active liquid metal solutions (SCALMS) are a new class of supported liquid phase catalysts composed of catalytically active liquid alloy droplets on a porous support. The alloys consist of a low-melting metal in which a smaller amount of a catalytically active metal, so far exclusively precious metals, is dissolved.^1^ In contrast to conventional supported liquid phase catalysis, the catalytic reaction in SCALMS occurs only at the highly dynamic liquid metal/gas interface, as the liquid metal does not provide any relevant solubility for the organic reactants. It has been shown for Ga–Pd SCALMS that the active metal is depleted from the interface and pops up as an active single atom to the surface during catalysis.^2,3^ The liquid nature of the supported alloy droplets under the reaction conditions has been confirmed through a combination of X-ray diffraction (XRD), scanning electron microscopy (SEM), X-ray photoelectron spectroscopy (XPS), and MD calculations.^1,4,5^ SCALMS catalysts exhibit very interesting performance in alkane and cycloalkane dehydrogenation, in particular Ga–Pd,^1^ Ga–Rh,^4^ and Ga–Pt^5–7^ systems. A common feature of these dehydrogenation catalysts is that coking, the most important deactivation mechanism for high-temperature hydrocarbon chemistry under reductive conditions, is largely suppressed.^7–9^ Catalytic experiments, spectroscopic methods and DFT calculations have indicated that the reason for the low coke formation is the single-atom nature of the active metal in the liquid Ga matrix.^4,10^ In fact, SCALMS provide a neoteric and technical approach to single-atom catalysis, a concept that has recently attracted much interest in the catalysis community.^11–13^

Aimed to further explore the scope of the SCALMS concept, our group has explored reaction classes beyond alkane dehydrogenation. In this paper, we present our findings in the field of selective C–C-linkage reactions and combine these with the first account of base-metal SCALMS. The idea to explore ethylene oligomerization was inspired by the fact that most catalysts for this reaction are molecular in nature and that the oligomerization of lighter alkenes is of substantial industrial interest.^14–16^ While this process is well known to happen using homogeneous catalysts,^17–19^ examples of heterogeneous systems triggering this reaction are relatively rare. Mesoporous Ni-aluminosilicates and Ni-exchanged zeolites have been known to be promising heterogeneous catalyst systems.^16,20–22^ Also for these systems the reaction is reported to exclusively take place at single-atom sites.

In this contribution, we demonstrate the applicability and durability of Ga–Ni SCALMS catalysts for selective ethylene oligomerization to linear butenes. Ga–Ni SCALMS were prepared through a physical impregnation method of a silica support using ultrasonication to produce a Ga-isopropanol emulsion, as recently introduced by some of us.^5^ The Ga–Ni atomic ratio (Ga_*x*_Ni) has been determined using inductively coupled plasma atomic emission spectroscopy (ICP-AES) (for experimental details see the ESI†). The catalytic experiments were carried out in a continuously operated fixed-bed tubular reactor connected to an online gas chromatograph (GC) with a flame ionization detector (FID) (for technical details see the ESI†). The thermodynamic distribution for linear butene isomers was obtained from computational simulations using ASPEN Plus® (for computational details see the ESI†). According to the phase diagram, the Ga–Ni alloy system will form fully liquid alloys for temperatures and compositions above the liquidus line and partially liquid alloys below this line, as shown in Fig. 1. The relatively broad liquid regime of the Ga–Ni phase diagram enables reactions under milder conditions (<300 °C).

**Fig. 1:**
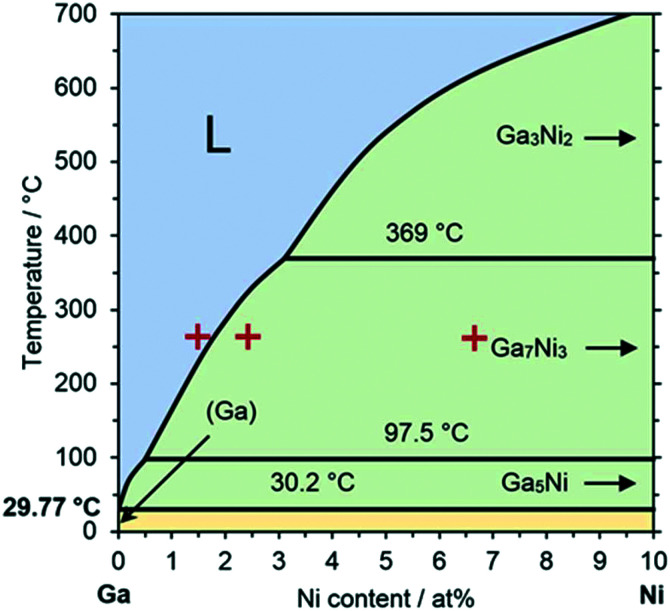
Phase diagram of the binary Ga–Ni metal alloy system up to 10 at% Ni in Ga. The blue area, denoted with “L”, above the liquidus line represents the liquid alloy regime, also referred to as the SCALMS regime. Systems in the green area form partially liquid alloy and solid intermetallic phases. The yellow area below 30.2 °C consists of mixed solid phases. The red crosses indicate the compositions applied for SCALMS in this work. Adapted from ref. 23.

Initial studies of the activity of SCALMS for oligomerization of ethylene were carried out using an alloy of 1.5 at% Ni in Ga supported on silica (Ga_67_Ni/SiO_2_) at 260 °C. Fig. 2 shows the ethylene conversion (*X*_ethylene_) and C4 alkene selectivities (*S*_C4-alkenes_) during the catalytic reaction for 24 h time on stream (TOS). The catalyst showed practically no initial activity followed by an activation period of approximately 10 hours until the conversion reached a stable plateau of around 7%. For the full operation time, the system demonstrated a high and relatively stable level of C4 alkene selectivity of 92%. The remaining products of the reaction were hexenes (C6) (6%) and octenes (C8) (2%), as expected from a Schulz–Flory distribution of the ethylene oligomerization process. The C4 products were found to be present as three isomers, namely, but-1-ene, *trans*-but-2-ene and *cis*-but-2-ene, in a 27/42/31 ratio. From ASPEN Plus® calculations, the thermodynamic equilibrium distribution of C4 isomers under the applied conditions is 17/48/35 showing that the mixture has approached almost the equilibrium composition. Assuming but-1-ene as the kinetic product of the oligomerization reaction, this C4 alkene distribution indicates that the isomerization reaction rate of the applied SCALMS systems is in a similar range to its oligomerization reaction rate under the here applied conditions. We hypothesize that the initial activation phase is due to the need to reduce a thin passivating Ga oxide skin on the Ga alloy droplets for reaching the full active SCALMS state of the system. Similar activation processes had been found in our previous studies of SCALMS catalysts in alkane dehydrogenation using Ga–Pt and Ga–Rh SCALMS reactions.^4–6^ There, it has been shown that the Ga-oxide skin can be efficiently reduced in the presence of a second metal in a reductive atmosphere either *in situ* or through hydrogen pretreatment. The passivating oxide skin on the Ga droplets is formed during the synthesis.

**Fig. 2:**
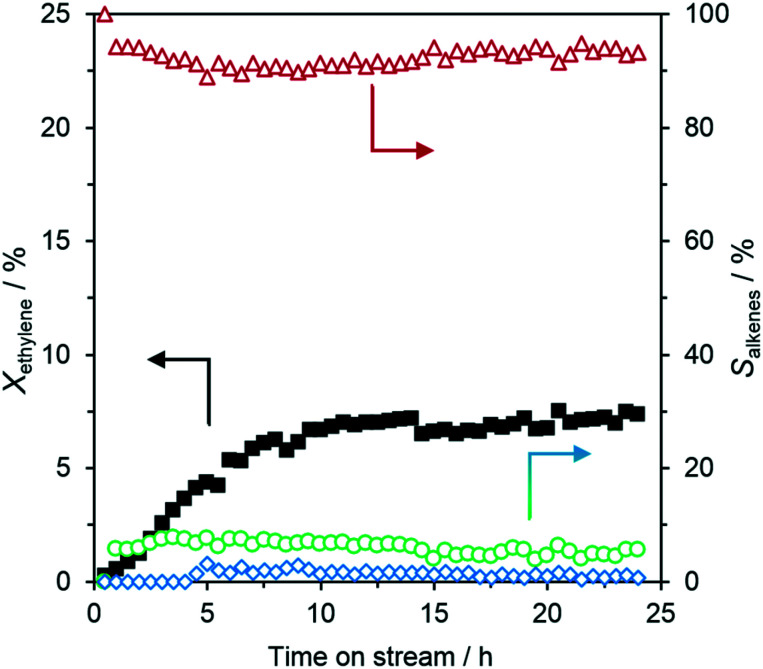
Ga–Ni SCALMS (Ga_67_Ni/SiO_2_) in the catalytic oligomerization of ethylene. Left axis: Ethylene conversion (*X*_ethylene_) (■), right axis: selectivity (*S*_alkenes_) for C4 (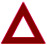), C6 (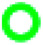), and C8 (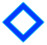) alkenes. Reaction conditions: 24 h on stream, 260 °C, 5 bar (g), 1.0 g_catalyst_, 3000 mL_N_ g_catalyst_^−1^ h^−1^ (GHSV = 490 h^−1^), 20 vol% C_2_H_4_ in He.

Although the oxide skin stabilizes the Ga alloy droplets from coalescing,^24^ this prevents the full catalytic activity of the supported liquid metal system. We interpret the induction phase of the Ga–Ni system as an indication that the system is able to self-reduce the Ga oxide skin at the supported Ga–Ni droplets at least to some degree, although the underlying mechanism has not been elucidated in detail yet. As no hydrogen is present during ethylene dimerization, the most likely scenario involves ethylene as a reducing agent. Temperature programmed reduction (TPR) also indicated reduction of the catalyst under a hydrogen stream (for details, see the ESI†).

To further confirm our hypothesis, we applied a dedicated reductive pretreatment to our Ga–Ni SCALMS material. Fig. 3 shows the performance of Ga_67_Ni/SiO_2_ after pretreatment with H_2_ at 310 °C for 2 h prior to the reaction under otherwise identical reactions conditions to those in the experiment without pretreatment. The positive effect of the hydrogen pretreatment can clearly be seen. The SCALMS system shows its full activity after the pretreatment without any additional activation process. The initial conversion is just above 12%, while the initial selectivities for C4, C6 and C8 are equal to the values obtained for the experiment without H_2_ pretreatment, *i.e.* 92% C4, 6% C6 and 2% C8 alkenes, respectively. This points to the fact that the pretreatment has only increased the availability of catalytically active surface, without changing the fundamental nature of the active sites formed. Compared to the non-pretreated performance, the level of stable conversion increased by more than 70%. Obviously, the pretreatment with H_2_ is able to activate a far greater reactive liquid metal interface than the self-activation process. Based on these findings, we decided to apply a distinct pretreatment process for all our subsequent ethylene oligomerization experiments, unless noted otherwise.

**Fig. 3:**
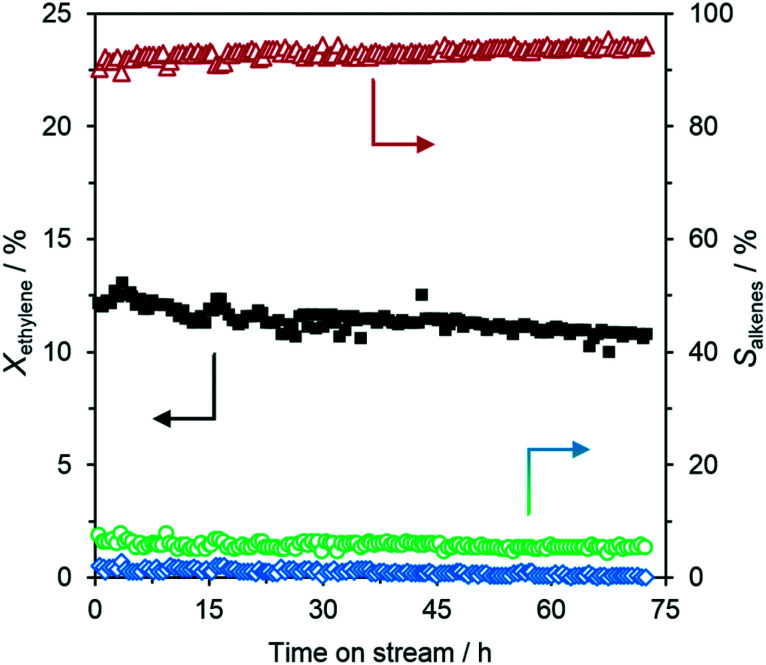
Long-term assessment of Ga–Ni SCALMS (Ga_67_Ni/SiO_2_) with H_2_ pretreatment in the catalytic oligomerization of ethylene. Left axis: Ethylene conversion (*X*_ethylene_) (■), right axis: selectivity (*S*_alkenes_) for C4 (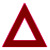), C6 (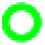), and C8 (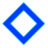) alkenes. Reaction conditions: 72 h on stream, 260 °C, 5 bar (g), 1.0 g_catalyst_, 3000 mL_N_ g_catalyst_^−1^ h^−1^ (GHSV = 490 h^−1^), 20 vol% C_2_H_4_ in He. Pretreatment: 2 h, 310 °C, 1200 mL_N_ g_catalyst_^−1^ h^−1^, 100 vol% H_2_.

The experiment shown in Fig. 3 was also intended to study the long-term stability of the Ga–Ni SCALMS system. Therefore, the catalyst was left on stream for 72 h in total and showed a quite stable performance during this period. The overall conversion marginally, but steadily, decreased from 12% to just below 11% after 3 day TOS. Concurrently, the selectivity towards C4 increased to 95% while the selectivity to C6 and C8 compounds decreased from 6% to 5% and 2% to 0%, respectively. Still, after 72 h TOS, the total selectivity to ethylene oligomerization products was found to be 100%. To identify the catalytically active species in our Ga–Ni SCALMS material, a series of reference materials were investigated under identical reaction conditions. The material compositions (determined by ICP-AES), as well as the catalytic performance of the different catalyst systems, are presented in Table 1. Besides the ethylene conversion and C4 selectivity also the Ni-based C4 productivity has been calculated for the different catalysts to allow better comparison. The pure SiO_2_ support (entry 1) showed no detectable activity for ethylene oligomerization at 260 °C and 5 bar (g) ethylene pressure. Supported Ga (entry 2) showed no activity, and supported Ga_2_O_3_ (entry 3) resulted in a minor initial activity that faded out within a few hours. To check whether supported Ni nanoparticles or NiO can act as an ethylene oligomerization catalyst on silica under the here applied conditions, we prepared the corresponding Ni on the silica material (for technical details see the ESI†) and applied it with and without pre-reduction with hydrogen (entries 4 and 5). As both systems showed negligible activity, we concluded that the observed oligomerization activity of the Ga–Ni SCALMS system is the result of the synergistic formation of the catalytic sites from Ni and Ga.

**Table 1:** Ethylene oligomerization using Ga–Ni SCALMS catalysts and reference materials^a^

Entry	Active material^b^	Ga loading^c^	Ni loading^c^	Ga/Ni ratio	*X*_ethylene_		*S*_C4-alkenes_		*P*_C4-alkenes_	
					3 h	40 h	3 h	40 h	3 h	40 h
		wt%	wt%	mol_Ga_ mol_Ni_^−1^	%		%		g_butene_ g_Ni_^−1^ h^−1^	
1	SiO_2_	—	—	—	<0.1	<0.1	—	—	—	—
2	Ga	14.4	—	∞	<0.1	<0.1	—	—	—	—
3	Ga_2_O_3_	2.85	—	∞	0.1	<0.1	100	—	—	—
4	Ni	—	0.52	0	<0.1	<0.1	—	—	<1	<1
5	NiO^d^	—	0.52	0	<0.1	<0.1	—	—	<1	<1
6	Ga_41_NiO_*x*_	3.16	0.06	41	0.6	0.6	100	100	6.5	6.5
7	Ga_15_Ni	1.21	0.07	15	2.2	1.4	94.4	94.8	20.2	12.4
8	Ga_41_Ni	2.18	0.05	41	6.2	5.3	93.4	93.5	87.1	75.2
9	Ga_67_Ni	3.34	0.04	67	12.5	11.4	92.2	93.1	196	181

a *X*_ethylene_ = ethylene conversion, *S*_C4-alekene_ = C4 selectivity, *P*_C4-alkenes_ = C4 productivity. Reaction conditions: 40 h on stream, 260 °C, 5 bar (g), 1.0 g_catalyst_, 3000 mL_N_ g_catalyst_^−1^ h^−1^ (GHSV = 490 h^−1^), 20 vol% C_2_H_4_ in He. Pretreatment: 2 h, 310 °C, 1200 mL_N_ g_catalyst_^−1^ h^−1^, 100 vol% H_2_.

b All catalysts were supported on SiO_2_.

c Determined experimentally using ICP-AES.

d Same as entry 4, but not pretreated with H_2_ prior to reaction.

This combination, as a function of the Ga/Ni ratio and the applied reaction temperature, can result in either supported solid Ga–Ni intermetallic compounds or – as characteristic for SCALMS systems – supported liquid alloy droplets. To further elucidate this structural difference, a mixed oxide of Ga_2_O_3_-NiO with a Ga/Ni-ratio of 41 (Ga_41_NiO_*x*_) (entry 6) was synthesized and tested. This mixed oxide phase is supposed to remain solid during the pretreatment with H_2_, while local reduction of the Ga oxide surrounding the Ni atoms at the interface is expected.^24^ The catalytic experiment resulted in low but constant activity (0.6% ethylene conversion; S_C4-alkenes_ = 100%; equilibrium distribution of C4 alkenes). To explore the catalytic performance of the Ga–Ni SCALMS system in more detail, three Ga–Ni compositions were prepared with different Ga/Ni ratios of 15, 41 and 67. Very similar Ni loadings and increasing Ga loadings were applied to adjust the respective ratios. The catalytic results are shown in entries 7, 8 and 9 of Table 1, respectively. The Ni-based activity was observed to increase significantly with higher Ga/Ni ratios, *i.e.* higher dilution of Ni in Ga leads to a more active system.

In our previous study on propane dehydrogenation using Ga–Rh SCALMS,^4^ this effect was already observed: the catalytic activity of a supported metal alloy shows a step-change in activity at the temperature or composition where the supported alloy crosses the liquidus line and fully liquid alloy droplet are present on the support. Estimated from the Ga–Ni phase diagram,^23^ the temperatures at which the alloys Ga_67_Ni, Ga_41_Ni and Ga_15_Ni cross the liquidus line are 230 °C, 280 °C and 570 °C, respectively. This means that under reaction conditions at 260 °C, only Ga_67_Ni is expected to form a fully liquid alloy. The other two systems may form some Ga-rich liquid parts but in this case, a significant amount of Ni is – according to the phase diagram – part of supported solid intermetallic compounds. Thus, the most active Ga–Ni catalyst system is indeed the one that represents a fully liquid SCALMS system (entry 9). Its initial Ni-based productivity of 196 g_butene_ g_Ni_^−1^ h^−1^ is more than four times higher than the Ga_41_Ni one, and almost 10 times higher than the Ga_15_Ni one. We conclude that also for the Ga–Ni system a SCALMS effect exists in the application of ethylene oligomerization, *i.e.* a drastic boost in Ni-based productivities is observed at the transition to a supported fully liquid alloy.

Table 1 also shows the long-term stability of the various Ga–Ni catalysts over 40 h time-on-stream. Comparing the initial Ni-based productivity values after 3 h with those after 40 h on stream, all catalyst materials under investigation show a slight decrease in productivity. However, the relative decrease is smallest for the Ga_67_Ni SCALMS (−7.6% compared to −13.7% and −38.6% for Ga_41_Ni and Ga_15_Ni, respectively). We propose that the better stability of the fully liquid SCALMS system can be attributed to the highly dynamic nature of the SCALMS liquid metal/gas interface as described previously for the case of precious metal SCALMS in alkane dehydrogenation catalysis.^7,8^ Thus, the here presented Ga–Ni SCALMS system shows great promise for future academic and industrial alkene oligomerization studies. From an academic perspective, a better fundamental understanding of the Ga–Ni SCALMS gas–liquid interface and its reactivity is desired. From an industrial perspective, the conversion of higher alkenes, such as propene and 1-butene, is of great interest, focusing on the degree of branching that is found in such higher alkene oligomerization reactions. It will be interesting to see how the special SCALMS nature of the reactive interface will affect the branching index of the obtained dimer products.

In conclusion, we have introduced here the application of non-precious metal SCALMS systems in catalysis by applying the Ga–Ni system successfully for ethylene oligomerization reactions. This example broadens the application field of SCALMS catalysts to selective C–C linkage reaction and breaks new ground for future studies using similar systems and transformations. In detail, our work has shown that the Ga–Ni SCALMS system provides a stable and efficient catalyst for the oligomerization of ethylene with a high selectivity towards C4 compounds, good Ni-based productivity and very interesting long-term stability.

## Conflicts of interest

There are no conflicts to declare.

## Supplementary Material

CY-011-D1CY01146D-s001
