# Supplementary material for: Ga–Ni supported catalytically active liquid metal solutions (SCALMS) for selective ethylene oligomerization
Source: Catal Sci Technol. 2021 Nov 15;11(23):7535–9. doi: 10.1039/d1cy01146d (PMC8630613; doi:10.1039/d1cy01146d)
Supplement: CY-011-D1CY01146D-s001 [file CY-011-D1CY01146D-s001.pdf]

Electronic Supplementary Material (ESI) for Catalysis Science & Technology.
This journal is © The Royal Society of Chemistry 2021

Supporting Information:

Ga-Ni Supported Catalytically Active Liquid Metal Solutions (SCALMS) for Selective Ethylene Oligomerization

Alexander Søgaard,[a] Ana Luísa de Oliveira,[b] Nicola Taccardi,[a] Marco Haumann[a] and Peter Wasserscheid*[a,b]

a) Friedrich-Alexander-Universität Erlangen-Nürnberg (FAU), Lehrstuhl für Chemische Reaktionstechnik (CRT), Egerlandstr. 3, 91058 Erlangen, Germany.

b) Forschungszentrum Jülich GmbH, Helmholtz-Institute Erlangen-Nürnberg for Renewable Energy (IEK 11), Egerlandstr. 3, 91058 Erlangen, Germany.

*Corresponding author. E-mail address: peter.wasserscheid@fau.de

Preparation of catalysts

Ga@SiO$_2$: The supported Ga catalyst (entry 2) were prepared through a physical deposition method using ultrasonication, similar to the procedure recently described by our group.[1] A 6 mm Gallium nugget (99.999%, Alpha-Aesar) of approximately 0.8 - 0.9 g was weighed and added to 100 mL propan-2-ol (techn. grade). The mixture was emulsified using ultrasonication (Branson 450 sonicator, 80% intensity for 10 min). After the sonication, an appropriate amount of silica support (spherical SiO_2 particles, Ø 75-200 µm, Sigma-Aldrich) (aiming for a 20 wt% Ga loading) was added to the mixture, and the solvent was slowly evaporated using a vacuum rotary evaporator. The resulting solid was calcined at 550 °C overnight.

Ga_2O_3@SiO_2: The supported Ga_2O_3 catalyst (entry 3) was prepared *via* impregnation of $Ga(NO_3)_3 \cdot xH_2O$ (gallium(III) nitrate hydrate, Puratronic®, 99.999%, Alfa Aesar) on SiO_2 support, drying and calcination of the final solid at 550 °C overnight.

Ni@SiO_2: The supported Ni catalyst (entries 4 and 5) was prepared *via* impregnation of $Ni(NO_3)_2 \cdot 6H_2O$ (nickel(II) nitrate hexahydrate, AnalaR NORMAPUR®, 99.0-102.0%, VWR Chemicals) on SiO_2 support, drying and calcination of the final solid at 550 °C overnight.

Ga_2O_3-NiO@SiO_2: The supported Ga_2O_3-NiO ($Ga_{41}Ni/SiO_2$) mixed oxide catalyst (entry 6) was prepared via impregnation of a mixture of $Ga(NO_3)_3 \cdot xH_2O$ and $Ni(NO_3)_2 \cdot xH_2O$ in a molar ratio of 50:1 on SiO_2 support, drying and calcination of the final solid at 550 °C overnight

Ga_xNi@SiO_2: The Ga-Ni SCALMS catalysts (entries 7, 8 and 9) were prepared by a physical deposition method. A 6 mm Gallium nugget (99.999%, Alpha-Aesar) of approximately 0.8 - 0.9 g was weighed and added to 100 mL isopropanol (techn. grade). The mixture was emulsified using ultrasonication (Branson 450 sonicator, 80% intensity for 10 min). An appropriate amount of the Nickel precursor (dichloro(dimethoxyethane)nickel(II), 98%, Sigma-Aldrich) was dissolved in 20 mL 1:9 water:propan-2-ol mixture before being added to the Ga emulsion and sonicated a second time (20 % intensity at 10 min). After the sonication, an appropriate amount of silica support (spherical SiO_2 particles, Ø 75-200 µm, Sigma-Aldrich) (aiming for a 7 wt% Ga loading) was added to the mixture, and the solvent was slowly evaporated using a vacuum rotary evaporator. The resulting solid was calcined at 550 °C overnight.

Elemental analysis and Ga/Ni-ratio evaluation

The Ga and Pt loading of the prepared catalysts was determined by inductively coupled plasma atomic emission spectroscopy (ICP-AES) using a Ciros CCD (Spectro Analytical Instruments GmbH). The solid samples were digested in 3:1:1 volumetric ratio of concentrated HCl:HNO_3:HF using microwave heating up to 220 °C for 40 min (CAUTION: HF is extremely harmful, relevant safety precautions must be taken). The instrument was calibrated for Ni (231.604 nm) and Ga (417.206 nm) with standard solutions of the particular elements prior to the analyses.

Catalytic experiments of ethylene oligomerization

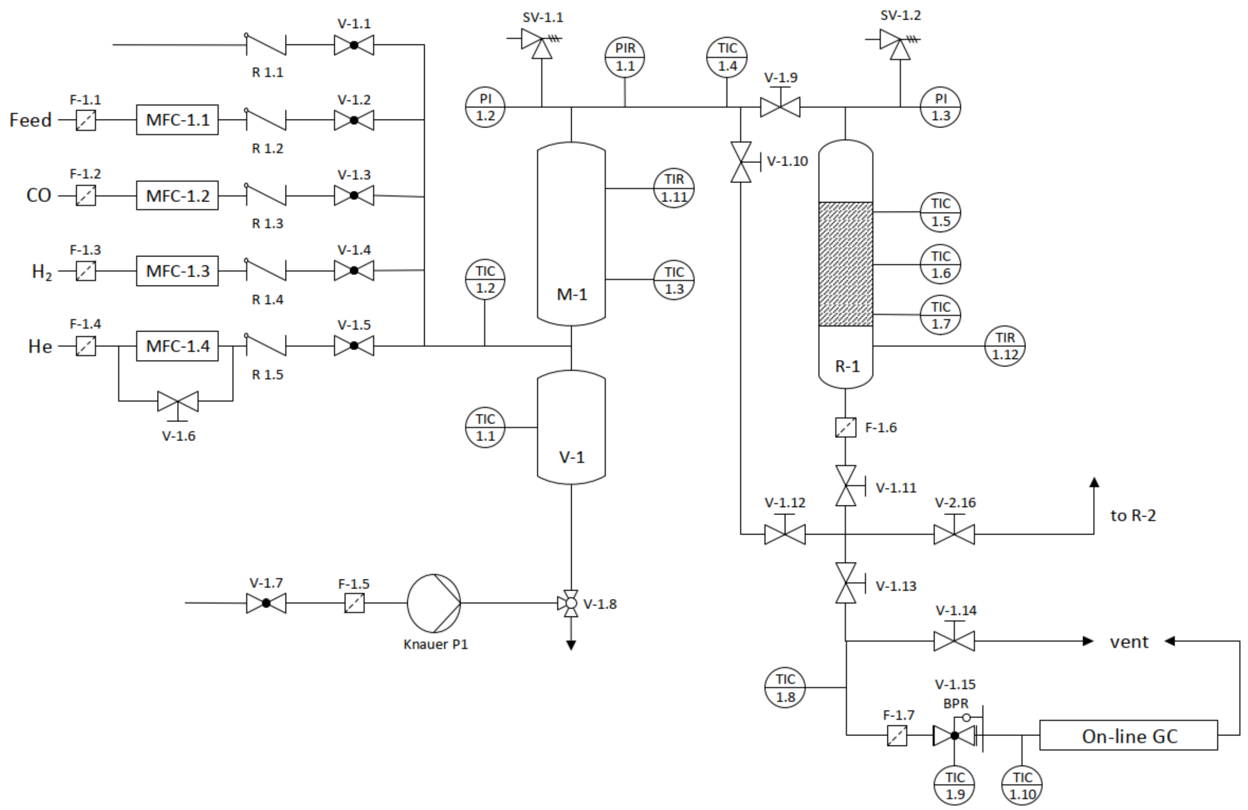

Figure S1: Schematical representation of the tubular reactor setup used for the catalytic oligomerization of ethylene.

The catalytic experiemnts were performed in a continuous tubular fixed-bed reacor setup. A schematic illustration of the plant setup is shown in Figure S1. Electical heating lines and inlet

flows were controlled remotely using LabVIEW software on a local PC. The inlet flows were controlled by mass flow controllers (MFCs) from Bronkhorst® EL-FLOW series. The inlet gas was mixed in the mixer (M-1) before being sent through the reactor (R-1), alternatively the by-pass. R-1 was heated by a Horst HMT electrical heating jacket. The pressure was increased by means of a back pressure regulator (BPR).

The product stream was analyzed by gas chromatography using a Agilent 7890A on-line GC equipped with two GC coulums in casade (a CP-WAX (25 m x 0.330 mm) and a Gas Pro (30 m x 0.320 mm)) and a flame ionization detector (FID).

Mole fractions x_i are calculated from peak areas and calibration factors determined for every substance. The conversion of ethylene (X_{ethylene}) (1), the selectivities (S_i) (2) and the C4 productivity ($P_{\text{C4-alkenes}}$) (3) were calculated as follows:

Conversion: $$X_{ethylene} = (1 - x_{C2}) \cdot 100\% \tag{1}$$

Selectivity: $$S_i = \frac{x_i}{x_{C4} + x_{C6} + x_{C8}} \tag{2}$$

Productivity: $$P_{C4-alkenes} = \left(\dot{n}_{C2} \cdot (X_{C2} \cdot S_{C4} \cdot M_{C4}) \cdot \frac{\nu_{C4}}{\nu_{C2}}\right) \cdot \frac{1}{m_{Ni}} \tag{3}$$

ASPEN Plus® simulations for thermodynamic butene isomer distribution

The isomerisation of C4 products of an ethylene oligomerisation was simulated using the chemical process software Aspen Plus® (version V10). The objective of these simulations were to determine the equilibria between the three structural isomers but-1-ene, *trans*-but-2-ene and *cis*-but-2-ene, allowing for comparison between experimentally obtained values and thermodynamic predictions.

Predictive Redlich Kwong-Soave (PSRK) property method was chosen following the guidelines of the Aspen Plus® manual. PSRK is recommended for gas-processing, refinery and petrochemical applications. Additionally, test simulations were also conducted over a wide range of temperatures (0-500 °C) and overall pressures (1-20 bar).

The process flowsheet comprised one main feed stream containing pure but-1-ene that was directed to an "RGibbs" type reactor, which is based on the minimisation of Gibbs free energy approach in convergence calculations, as shown in Figure S2. No reaction equations were provided. Instead, only expected compounds for various assumptions were defined in the outlet stream of the reactor, namely but-1-ene, *trans*-but-2-ene and *cis*-but-2-ene.

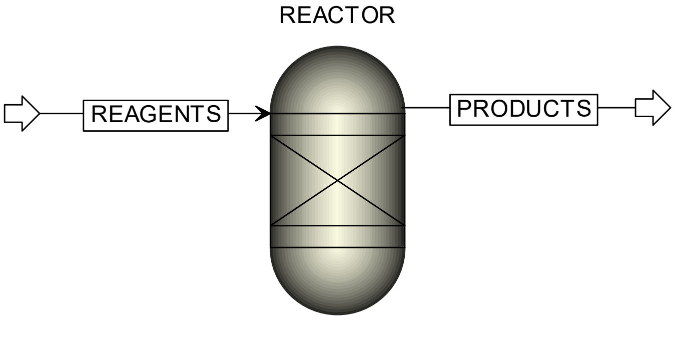

Figure S2. Main flowsheet of the simulation performed in Aspen Plus software. The feed stream consists of pure but-1-ene at 260 °C and 6 bar flowing into an RGibbs type reactor at 2 kmol h^{-1}. The reactor operates at 260 °C and 6 bar. Here all potential reactions involving but-1-ene, trans-but-2-ene and cis-but-2-ene can take place.

Temperature Programmed Reduction (TPR) of Ga-Ni SCALMS catalyst

To investigate the reduction phenomena during H_2 pretreatment of the Ga-Ni SCALMS catalyst, temperature programmed reduction (TPR) were performed. The reduction of fresh $Ga_{67}Ni/SiO_2$ under 20% H_2 in Ar up to 500 °C are shown in Figure S3.

An uptake of hydrogen is observed already from room temperature (RT) until approximately 200 °C, with a small boost in uptaking at 55°C. Between 200 °C and approximately 400 °C the consumption of hydrogen is more or less stable. Above 400 °C, the TCD signal decreases until the measurement reaches the isothermal at 500 °C at which point, the signal remains constant.

During the measurement, no distinctive peaks are observed. Although, with the limited amount of Ga oxide present on the surface of the Ga-Ni droplets, large reduction peaks would not have been expected. Different sorption phenomena, such as physisorption and chemisorption, are all likely to overlap with any potential reduction peaks. However, with these results showing an uptake of hydrogen until 200 C° and according to the data from to literature[2, 3], and previous investigations of SCALMS catalysts[4, 5], it is reasonable to assume that reduction of the Ga oxide skin is taking place under the applied pretreatment conditions.

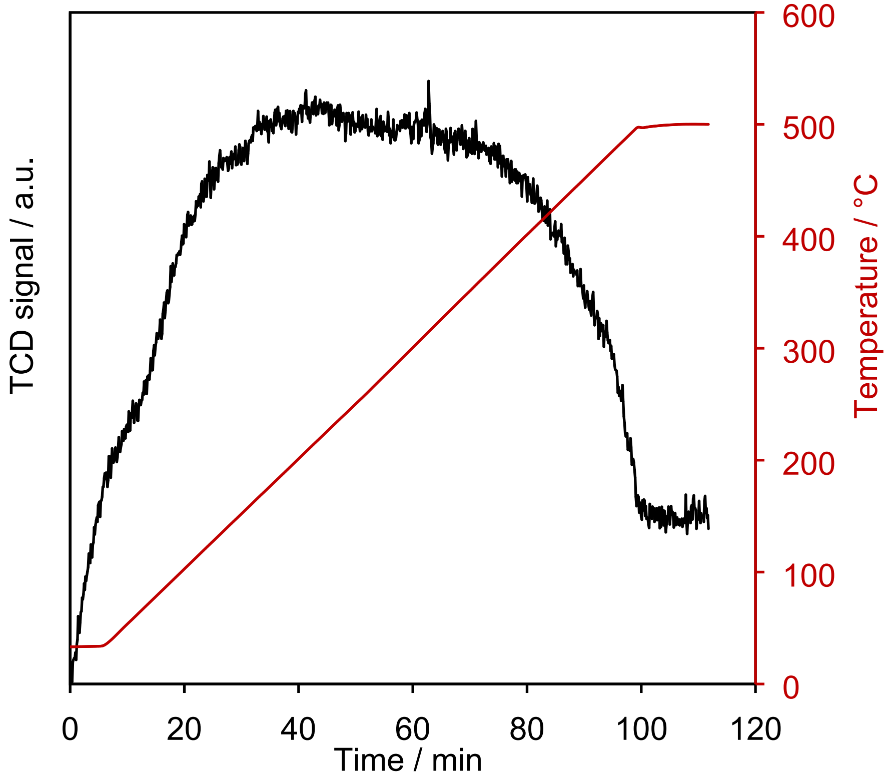

Figure S3: Temperature Programmed Reduction (TPR) of $Ga_{67}Ni/SiO_2$. Black line = TCD signal. Red line = temperature profile of the experiment. Experimental conditions: 50 mL_N min^{-1}, 20% H_2 in Ar, RT to 500 °C, 5 K min^{-1}, isotherms: 5 min pre-ramp, 10 min post-ramp, sample weight = 300.6 mg.

References

1. N. Raman, M. Wolf, M. Heller, N. Taccardi, M. Haumann, P. Felfer and P. Wasserscheid, *ACS Catal*, 2021, **Submitted**.
2. A. Gallo, J. L. Snider, D. Sokaras, D. Nordlund, T. Kroll, H. Ogasawara, L. Kovarik, M. S. Duyar and T. F. Jaramillo, *Applied Catalysis B: Environmental*, 2020, **267**, 118369.
3. M. Wencka, J. Kovač, V. D. B. C. Dasireddy, B. Likozar, A. Jelen, S. Vrtnik, P. Gille, H. J. Kim and J. Dolinšek, *Journal of Analytical Science and Technology*, 2018, **9**.
4. N. Raman, S. Maisel, M. Grabau, N. Taccardi, J. Debuschewitz, M. Wolf, H. Wittkamper, T. Bauer, M. Wu, M. Haumann, C. Papp, A. Gorling, E. Spiecker, J. Libuda, H. P. Steinruck and P. Wasserscheid, *ACS Catal*, 2019, **9**, 9499-9507.
5. O. Sebastian, S. Nair, N. Taccardi, M. Wolf, A. Søgaard, M. Haumann and P. Wasserscheid, *ChemCatChem*, 2020, **12**, 4533-4537.
